# Neural mechanism of *Shugan Jieyu* combined with zolpidem in insomnia disorder with depressive symptoms: evidence from event-related potentials in Go/No-Go and psychomotor vigilance task

**DOI:** 10.1186/s12888-026-08023-y

**Published:** 2026-03-28

**Authors:** Qianqian Xin, Dhirendra Paudel, Jiehan Zhang, Huafeng Wei, Haokang Huang, Zhihong Lv, Wenjuan He, Yan Xu, Bin Zhang

**Affiliations:** 1 Department of Psychiatry, Sleep Medicine Center, Nanfang Hospital, Southern Medical University, No 1838, Guangzhou Northern Road, Guangzhou, Guangdong 510515 China; 2 Key Laboratory of Mental Health of the Ministry of Education, Southern Medical University, Guangzhou, Guangdong 510515 China; 3 Guangdong-Hong Kong-Macao, Greater Bay Area Center for Brain Science and Brain-Inspired Intelligence, Southern Medical University, Guangzhou, Guangdong 510515 China; 4 Shanxi Datong University School of Medicine, Datong, Shanxi 037009 China

**Keywords:** Insomnia, Depression, Response inhibition, Event-related potential

## Abstract

**Objective:**

The study aims to investigate the combination’s effects of *Shugan Jieyu* and zolpidem on executive functions and sustained vigilance in patients with Insomnia Disorder with Depressive Symptoms (IDDS).

**Methods:**

In this randomized controlled trial, IDDS patients were allocated to receive either zolpidem plus *Shugan Jieyu* (ZS group) or zolpidem plus placebo (ZP group) for 8 weeks. Go/No-Go and Psychomotor Vigilance Task (PVT), coupled with Electroencephalography (EEG), were used to assess behavioral performance and underlying neurophysiological activity at baseline, fourth week, and eighth week after treatment.

**Results:**

While no significant between-group differences were found in the behavioral measures of the Go/No-Go task, an exploratory ERP analysis revealed that the ZS group showed a more negative Go N2 amplitude at the fourth week compared to the ZP group, F(1,56) = 5.161, *p* = 0.027, suggesting a potential early enhancement of conflict monitoring. In the PVT, the ZS group demonstrated significantly faster mean reaction times at the eighth week (F(1,57) = 4.143, *p* = 0.046). For attention lapses, no significant Group × Time interaction was found (F(1.834,104.543) = 0.515, *p* = 0.583); however, an exploratory analysis suggested fewer lapses in the ZS group at Week 8 (F(1,57) = 4.301, *p* = 0.043). For ERP, a significant Group × Time interaction was found for the N1 amplitude (F(1.829, 104.242) = 3.314, *p* = 0.044), indicating that N1 negativity increased over time in the ZP group but remained stable in the ZS group, suggesting a potential stabilization of early sensory processing.

**Conclusions:**

The combination of *Shugan Jieyu* and zolpidem was associated with cognitive function improvements in patients with IDDS, including enhanced neural conflict monitoring and improved sustained vigilance. These findings suggest that this combination therapy may offer cognitive benefits beyond sleep improvement in IDDS.

**Trial Registration:**

ClinicalTrials.gov (NCT05764798), registered on February 19, 2023.

## Introduction

The estimated prevalence of insomnia in the population ranges from 10% to 60% [6]. It frequently co-occurs with depressive symptoms and is clinically conceptualized as Insomnia Disorder with Depressive Symptoms (IDDS) [25, 37, 38]. Current treatment strategies for IDDS often involve a combination of pharmacotherapy and psychotherapy. Antidepressants, such as selective serotonin reuptake inhibitors (SSRIs), are commonly used for depressive symptoms but can have delayed onset and side effects that may initially worsen insomnia [55]. Hypnotics like zolpidem, a non-benzodiazepine GABA_A_ receptor positive allosteric modulator, are effective for sleep initiation but do not directly address mood or the full spectrum of daytime cognitive deficits [47]. While Cognitive Behavioral Therapy for Insomnia (CBT-I) is a highly effective first-line treatment, access remains limited [42]. Consequently, a significant treatment gap exists for interventions that can simultaneously improve sleep, mood, and cognitive function in IDDS.

In addition to this treatment gap, the bidirectional relationship between the two is well-established, and research has shown that treating depression alone is often insufficient to resolve the primary insomnia [7, 12], while the persistence of insomnia can aggravate and maintain depressive symptoms [39]. Therefore, effective interventions for IDDS must target the insomnia directly while simultaneously managing depressive symptoms to improve sleep and prevent their exacerbation [29, 43]. Beyond the core clinical symptoms, IDDS is strongly associated with significant daytime cognitive impairment [9, 14, 20, 21, 35]. This cognitive impairment stems from the combined deficits associated with both insomnia and depressive symptoms. On one hand, difficulty concentrating is one of the most common complaints in insomnia, potentially reflecting deficits in sustained and selective attention [1, 46]. On the other hand, depressive symptoms are linked to cognitive dysfunction, particularly in the domains of attention, executive function, and psychomotor speed [14].

Studies consistently report that individuals with insomnia exhibit poorer performance on attention tasks, including reduced accuracy and slower processing speeds, which can impact daily functioning such as work performance [2, 24, 30, 54]. Furthermore, insomnia impacts higher-order executive functions, which are responsible for processes such as planning, reasoning, inhibitory control, cognitive flexibility, and multitasking [33, 48]. A meta-analysis confirmed cognitive deficits of small to moderate magnitude in individuals with insomnia, particularly evidenced by slower reaction time (RT) on tasks measuring inhibitory control and cognitive flexibility [4]. Similarly, systematic reviews on depression have demonstrated moderate deficits in executive function, memory, and attention in patients with depressive symptoms compared to healthy controls [22]. There is growing evidence that these cognitive symptoms (e.g., concentration and memory problems) play a significant role in functional outcomes [23, 45]. The “vigilance attention hypothesis” posits that deficient alertness may serve as a core mechanism underlying broader cognitive impairments [16, 32]. While this hypothesis has existed for years, few studies have explicitly tested the relationship between vigilance and executive functions, such as response inhibition, in the IDDS population [50, 51].

Addressing this gap in the literature requires novel therapeutic approaches. Traditional Chinese Medicine offers potential adjunctive treatments. *Shugan Jieyu*, a compound preparation of *Hypericum perforatum* (St. John’s Wort) and *Acanthopanax senticosus*, is approved in China for mild-to-moderate depression. Its proposed mechanism involves the modulation of monoaminergic neurotransmitters (serotonin, dopamine, norepinephrine), similar to conventional antidepressants, which may contribute to its mood-regulating effects and daytime cognitive improvement [11, 34]. We hypothesized that combining *Shugan Jieyu* with zolpidem would provide synergistic benefits for cognitive function in IDDS. By improving mood and reducing hyperarousal via monoaminergic modulation, *Shugan Jieyu* might create a more favorable neural state for cognitive recovery, while zolpidem directly improves sleep. This synergy could enhance cognitive processes such as conflict monitoring (indexed by the N2 event-related potential (ERP) component) and sustained vigilance (indexed by Psychomotor Vigilance Task (PVT) performance and the N1 and P3 ERP components), beyond the effects of zolpidem alone. This study aims to investigate the effects of the combination therapy of *Shugan Jieyu* and zolpidem on response inhibition and sustained vigilance in IDDS patients. We seek to determine whether the combination therapy confers specific advantages over zolpidem alone in enhancing these critical cognitive domains.

## Materials and methods

### Participants

Sixty adults participated in the experiment. The study cohort comprised IDDS patients identified through the outpatient department. Participant recruitment commenced on March 1, 2022, and all final assessments were completed by October 2023. All participants had normal or corrected-to-normal vision. Participant enrollment criteria were consistent with our primary analysis of insomnia severity [58].

The eligibility criteria for inclusion in this study were as follows: (1) a primary diagnosis of Insomnia Disorder according to Diagnostic and Statistical Manual of Mental Disorders, Fifth Edition (DSM-5) criteria; (2) co-occurring depressive symptoms, defined by a Patient Health Questionnaire-9 (PHQ-9) score ≥ 10, in the absence of a full Major Depressive Disorder (MDD) diagnosis; (3) age between 18 and 60 years; and (4) no recent therapy for insomnia or depression within the preceding month.

Key exclusion criteria comprised: (1) contraindications to the study medications or conditions affecting drug metabolism (e.g., specific allergies, hepatic conditions, substance use disorders); (2) participation in another clinical trial recently; (3) significant chronic physical or comorbid mental disorders; (4) other primary sleep disorders, specifically sleep apnea-hypopnea index (AHI ≥ 15) or periodic limb movement disorder (PLMI > 15); (5) night or shift work schedules; (6) pregnancy or lactation; and (7) significant suicidal risk.

Participants were randomly allocated (1:1) to one of two parallel groups for an 8-week, double-blind intervention: the experimental group (ZS) received *Shugan Jieyu* combined with zolpidem, while the control group (ZP) received zolpidem with a matched placebo. A computer-generated random number sequence was used for allocation. Of the 60 patients enrolled, 59 completed the trial (30 in the ZS group and 29 in the ZP group).

### Study design and procedure

This was an 8-week, double-blind, randomized controlled trial. Following enrollment and baseline assessments, participants were randomized to either the ZS or ZP group. Participants received zolpidem (10 mg once daily, taken 30 min before bedtime) and were also randomly assigned to receive either *Shugan Jieyu* or a matched placebo (0.72 g twice daily). All three assessment points (Baseline, Week 4, and Week 8) followed an identical procedure. On each assessment day, participants arrived at the sleep laboratory in the morning. They first completed clinical questionnaires, followed by the EEG setup. The Go/No-Go and PVT tasks were then administered in a counterbalanced order across participants. To avoid acute medication effects on the EEG measures, the morning assessment ensured participants had not taken their study medication for approximately 10–12 h (i.e., since the previous night’s dose).

### Go/No-Go task

The Go/No-Go task was employed to assess response inhibition. The Go/No-Go task was presented using E-Prime software. Participants were required to respond quickly to frequent “Go” stimuli while withholding their response to infrequent “No-Go” stimuli. Each trial began with a variable inter-stimulus interval of 1000 to 3000 ms, followed by the presentation of a Go (O or A) or No-Go stimulus (A or O) for 250 ms. The stimuli were presented in white on a black background. A blank black screen then appeared for 1500 ms. When the letter “O” appeared under the Go task, the participant was asked to press “1” with their right index finger as soon as possible, while when the letter “A” appeared under the No-Go task, the participant was asked to stop the motor response. There were 120 trials in this task, 75% of which were stimulated by Go and 25% by No-Go. Each participant practiced the cognitive task before the experiment to ensure that he/she fully understood the instructions. The Go/No-Go task serves as a well-validated paradigm for assessing this function, requiring participants to respond selectively to target stimuli while inhibiting responses to non-targets [17]. Within this framework, the N2 and P3 ERP components are widely regarded as neurophysiological indices of response inhibition and conflict monitoring [49].

### Psychomotor vigilance task (PVT)

Sustained vigilance was evaluated using the PVT. In this simple RT task, participants were instructed to respond as quickly as possible to a visual stimulus that appeared at random intervals. The PVT was a 10-minute task. A digital millisecond counter was presented on a blank screen, and participants were instructed to press a response button as soon as the counter started. The inter-stimulus interval varied randomly from 2 to 10 s. The primary behavioral outcomes were mean RT and lapses (responses slower than 500 ms). Concurrent ERPs recordings were analyzed, focusing on the N1 component related to early sensory processing and the P3 component associated with attentional resource allocation [57].

### EEG recordings and analysis

Data acquisition took place in an electromagnetically isolated environment. The EEG signal was captured via a 64-electrode setup (Neuroscan Curry8) conforming to the extended 10–20 layout, incorporating a prefrontal online reference. With all channels calibrated to an impedance below 10 kΩ, the data were sampled at 1000 Hz. The pre-processing pipeline, implemented in EEGLAB v2020.0, involved several steps: initial bandpass filtering (0.1–30 Hz), manual scrutiny to reject epochs with motion or signal dropouts, and finally, Independent Component Analysis-based correction to address blinks and EMG activity. Epochs were extracted from − 200 ms to 800 ms relative to stimulus onset, re-referenced to the average of the bilateral mastoids, and baseline-corrected using the 200 ms pre-stimulus interval. Epochs were then categorized by condition (e.g., Go vs. No-Go). Trials with voltage exceeding ± 150 µV were automatically rejected. Trials in which subjects made performance errors were also excluded.

In Go/No-Go, two ERP components, N2 and P3, were of interest in this study. The N2 was scored on stimulus-locked waves as the largest peak negative deflection with a frontocentral topography (averaged across values recorded at FCz, Fz, and Cz) between 200 and 400 ms after No-Go stimulus onset. The P3, a long waveform, was determined by taking the mean activation across parietal sites between 300 and 600 ms after the stimulus (average across values recorded at Pz, P3, and P4) (Fleck et al., 2019).

In PVT, RT > 500 ms and no response attempts were excluded. Two ERP components, N1 and P3, were of interest in this study. N1 amplitude was maximal on P7 and P8 and was quantified as the average amplitude in the 50–150 ms latency interval (averaged across values recorded at P7 and P8). The P3, a long waveform, was determined by taking the mean activation across parietal sites between 300 and 600 ms after the stimulus (average across values recorded at Pz, P3, and P4) [8].

For each ERP component, the amplitude was quantified as the peak positive or negative voltage within a specified time window, with the corresponding latency measured as the time from stimulus onset to that peak.

### Statistical analysis

All statistical analyses were performed using Statistical Package for the Social Sciences (SPSS) version 26.0 (IBM Corp., Armonk, NY, USA). The sample size of 60 was determined based on an a priori power analysis for a repeated-measures ANOVA (within-between interaction) aiming to detect a medium effect size (f = 0.25) with 85% power and an alpha level of 0.05, anticipating a 20% dropout rate.

Descriptive statistics are presented as mean ± standard deviation (SD) for continuous variables and as frequency (percentage) for categorical variables. Baseline demographic and clinical characteristics were compared between the ZS and ZP groups employing independent samples t-tests and Chi-square tests for continuous and categorical variables, respectively.

Prior to conducting the repeated-measures ANOVA, the assumptions of normality (assessed using Shapiro-Wilk tests) and sphericity (assessed using Mauchly’s test of sphericity; Greenhouse-Geisser correction applied when violated) were verified. For the primary analysis of Go/No-Go and PVT outcomes (both behavioral and ERP measures), a repeated-measures analysis of variance (ANOVA) was conducted with Time (Baseline, 4 Weeks, 8 Weeks) as the within-subjects factor and Group (ZS, ZP) as the between-subjects factor. The main effects of Time and Group, as well as the Time × Group interaction, were examined. The results of these analyses are presented in three tables, detailing the behavioral and electrophysiological outcomes for the Go/No-Go and PVT tasks.

When a significant Group × Time interaction was found, post-hoc comparisons were conducted using one-way ANOVA to examine group differences at each time point, with Bonferroni correction applied for multiple comparisons. Additionally, based on our a priori hypotheses regarding potential time-specific effects of the combination therapy, we conducted exploratory between-group comparisons at Week 4 and Week 8 using one-way ANOVA, regardless of the significance of the omnibus interaction. Given the exploratory nature of these analyses, the findings are presented with their uncorrected p-values and should be interpreted cautiously.

The significance level for all tests was set at *p* < 0.05.

## Results

A total of 60 participants were enrolled in the study. One participant in the ZP group withdrew from the trial, citing a slow response to the medication. All 30 participants in the ZS group completed the trial; however, one subject in the ZS group was excluded from the Go/No-Go analysis due to poor-quality EEG data. Consequently, 58 participants (ZS: *n* = 29; ZP: *n* = 29) for the Go/No-Go task and 59 participants (ZS: *n* = 30; ZP: *n* = 29) for the PVT were included in the final analysis.

The baseline sample characteristics of the two groups are presented in Table 1. The ZS and ZP groups were well-matched at baseline, showing no significant differences in age, sex, BMI, or education level (all *p* > 0.05).

**Table 1:** Baseline characteristics of patients in the ZS and ZP groups

Characteristic	Total	*n* (%) or mean ± SD		Statistics	*P*-value
		ZS group	ZP group		
Number of samples	59	30	29		
Age (years)	27.51 ± 6.69	28.50 ± 7.78	26.50 ± 5.28	1.325	0.267
Male	23 (39.0)	14 (46.7)	9 (31.0)	1.515	0.218
BMI, kg/m^2^	21.01 ± 2;91	21.42 ± 2.95	20.59 ± 2.84	1.223	0.273
Education (bachelor and above)	41 (69.5)	20 (66.7)	21 (72.4)	0.230	0.632
PHQ-9	15.44 ± 3.62	15.17 ± 3.21	15.72 ± 4.04	2.440	0.561
ISI	18.81 ± 4.00	18.70 ± 4.38	18.90 ± 3.64	1.600	0.826

Notes: ZS (Zolpidem + *Shugan Jieyu*); ZP (Zolpidem + Placebo); BMI = Body Mass Index; PHQ-9, Patient Health Questionnaire-9; ISI, Insomnia severity index; P-values are derived from independent samples t-tests for continuous variables (Age, BMI, ISI, PHQ-9) and Chi-square tests for categorical variables (Sex, Education)

### Go/No-Go task: behavioral and electrophysiological measures

Behavioral performance on the Go/No-Go task is summarized in Table 2. Repeated-measures ANOVA revealed no significant main effects of Group or Group × Time interactions for mean RT, Go trial accuracy, or No-Go trial accuracy (all *p* > 0.05). A significant main effect of Time was found for No-Go accuracy (*p* = 0.017), indicating that both groups improved their inhibitory accuracy over the course of the treatment.

**Table 2:** Go/No-Go parameters in patients with IDDS across the treatment period

Variable	Group	Baseline	4 Weeks	8 Weeks	Statistics	Post
Behavioral						
Mean RT, ms	ZS	0.40 ± 0.06	0.41 ± 0.05	0.41 ± 0.04	Fg(1, 56) = 0.985, *p* = 0.325, η²=0.017 Ft (1.335, 74.788) = 0.032, *p* = 0.916, η²=0.001 Fgt (1.335, 74.788) = 0.388, *p* = 0.596, η²=0.007	n.s.
	ZP	0.42 ± 0.09	0.42 ± 0.05	0.42 ± 0.06		
Go Accuracy, %	ZS	96.79 ± 4.62	96.99 ± 3.87	97.97 ± 3.59	Fg(1, 56) = 0.379, *p* = 0.540, η²=0.007 Ft(1.383, 77.477) = 1.135, *p* = 0.309, η²=0.020 Fgt(1.383, 77.477) = 0.072, *p* = 0.865, η²=0.001	n.s.
	ZP	96.10 ± 5.73	95.80 ± 12.68	97.55 ± 3.78		
No Go Accuracy, %	ZS	93.28 ± 6.67	94.66 ± 4.85	94.54 ± 6.38	Fg(1, 56) = 0.019, *p* = 0.892, η²<0.001 Ft(1.681, 94.133) = 4.622, p = **0.017**, η²=0.076 Fgt(1.681, 94.133) = 0.569, *p* = 0.539, η²=0.010	n.s.
	ZP	92.23 ± 7.86	94.65 ± 4.62	95.06 ± 5.17		
ERP Component Amplitude						
N2 - Go, µV	ZS	-3.44 ± 1.63	-3.66 ± 1.61	-3.83 ± 1.88	Fg=(1, 56) = 2.909, *p* = 0.094, η²=0.049 Ft=(1.979, 110.812) = 2.971, *p* = 0.056, η²=0.050 Fgt=(1.979, 110.812) = 1.488, *p* = 0.230, η²=0.026	ZS > ZP at 4 weeks¹ F(1, 56) = 5.161, p = **0.027**, η²=0.084
	ZP	-2.89 ± 2.06	-2.53 ± 2.14	-3.20 ± 1.98		
N2 – No Go, µV	ZS	-3.56 ± 1.71	-3.61 ± 1.48	-3.94 ± 1.65	Fg=(1, 56) = 1.762, *p* = 0.190, η²=0.031 Ft=(1.965, 110.013) = 3.763, p = **0.027**, η²=0.063 Fgt=(1.965, 110.013) = 0.281, *p* = 0.751, η²=0.005	n.s.
	ZP	-2.86 ± 2.20	-2.93 ± 2.46	-3.53 ± 1.97		
P3 - Go, µV	ZS	4.87 ± 1.99	5.13 ± 2.32	4.78 ± 2.01	Fg=(1, 56) = 0.665, *p* = 0.418, η²=0.012 Ft=(1.835, 102.741) = 1.216, *p* = 0.298, η²=0.021 Fgt=(1.835, 102.741) = 0.754, *p* = 0.462, η²=0.013	n.s.
	ZP	4.75 ± 2.17	4.51 ± 2.01	4.28 ± 2.34		
P3 – No Go, µV	ZS	3.51 ± 2.03	3.66 ± 1.89	3.11 ± 1.81	Fg=(1, 56) = 0.226, *p* = 0.637, η²=0.004 Ft=(1.997, 111.856) = 2.300, *p* = 0.105, η²=0.039 Fgt=(1.997, 111.856) = 1.518, *p* = 0.224, η²=0.026	n.s.
	ZP	3.55 ± 1.64	3.04 ± 1.90	3.06 ± 2.20		

Notes: Data are mean ± SD. Fg, Ft, and Fgt represent F-values for the Group, Time, and Group × Time interaction effects, respectively; RT, reaction time; ERP, Event-Related Potential; ZS: Zolpidem + *Shugan Jieyu* (*n* = 29); ZP: Zolpidem + Placebo (*n* = 29); n.s. = not significant. Greenhouse-Geisser corrected degrees of freedom and F-values are reported where Mauchly’s test of sphericity was violated (*p* < 0.05)

**Fig. 1:**
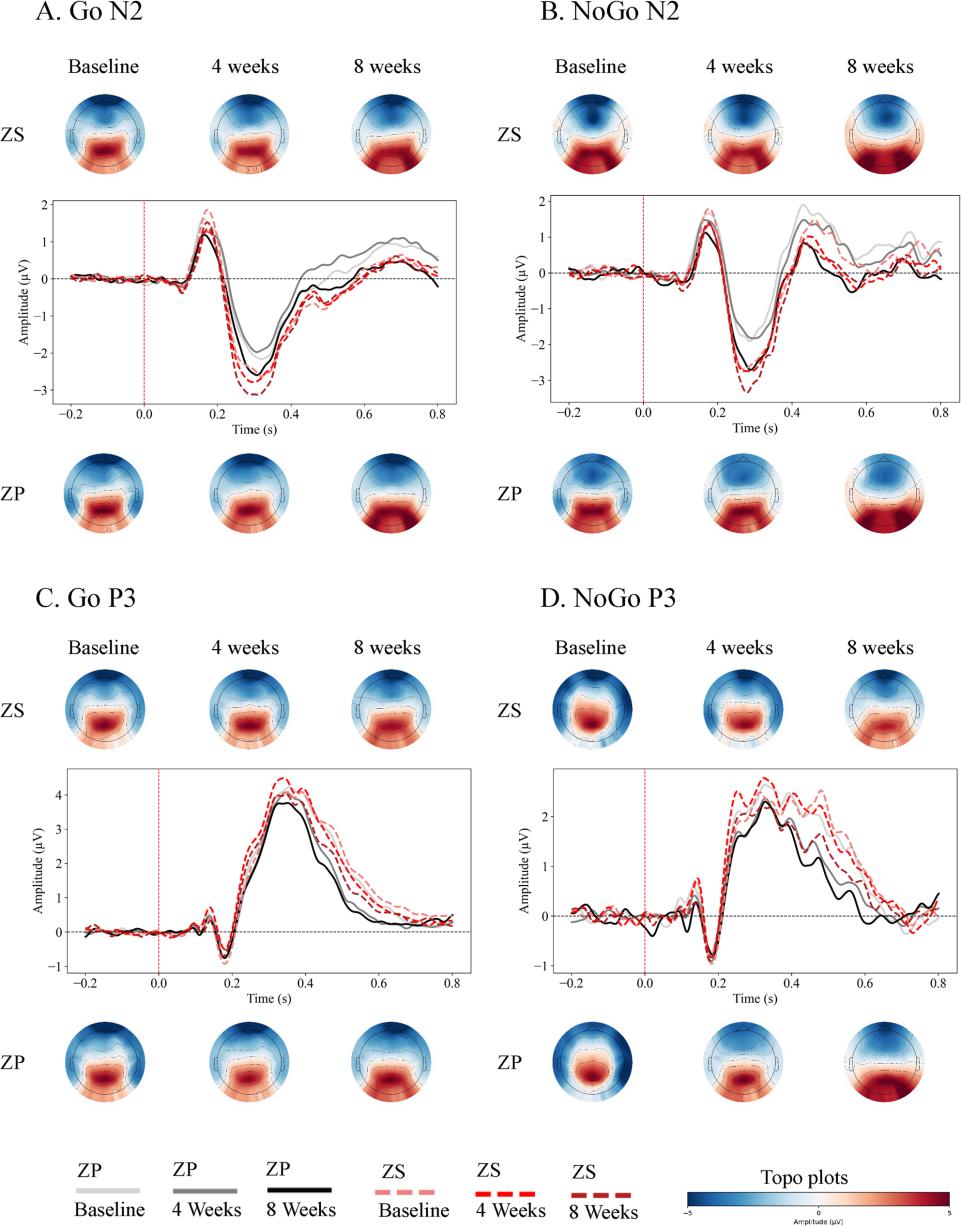
Event-Related Potential (ERP) waveforms and topographic maps for the Go/No-Go task across the treatment period. (**A**) Grand-average ERP waveforms for the N2 component on Go trials, and corresponding topographic maps of the N2 mean amplitude distribution (200–400 ms) for the ZS (Zolpidem + Shugan Jieyu) and ZP (Zolpidem + Placebo) groups at Baseline, 4 Weeks, and 8 Weeks. (**B**) Grand-average ERP waveforms and topographic maps for the N2 component on No-Go trials. (**C**) Grand-average ERP waveforms for the P3 component on Go trials, and topographic maps of the P3 mean amplitude distribution (300–600 ms). (**D**) Grand-average ERP waveforms and topographic maps for the P3 component on No-Go trials. The topographic maps illustrate the scalp voltage distribution, with blue representing negative potentials and red representing positive potentials

In contrast to the behavioral results, the ERP analysis revealed a trend toward an early neurophysiological effect of the combination therapy. For the N2 amplitude on Go trials, although the repeated-measures ANOVA did not reveal significant main effects of Group (F(1,56) = 2.909, *p* = 0.094) or Time (F(1.979, 110.812) = 2.971, *p* = 0.056), nor a significant Group × Time interaction (F(1.979, 110.812) = 1.488, *p* = 0.230), an exploratory ANOVA at the 4-week assessment suggested that the ZS group showed a more negative N2 amplitude compared to the ZP group (F(1, 56) = 5.161, *p* = 0.027). Given the non-significant omnibus results, this finding should be interpreted with extreme caution and considered only as a preliminary observation requiring confirmation in future studies. The grand-average ERP waveforms and topographic maps for the N2 and P3 components across all time points and conditions are presented in Fig. 1 (Fig. 1).

### Psychomotor vigilance task: behavioral and electrophysiological measures

The results for the PVT are detailed in Table 3. Behaviorally, the ZS group demonstrated superior performance at the end of the treatment period. For the lapses rate, no significant Group × Time interaction was found (F(1.834,104.543) = 0.515, *p* = 0.583). However, based on our a priori hypothesis regarding potential time-specific effects of the combination therapy, an exploratory between-group comparison at Week 8 suggested that the ZS group had a lower lapses rate (13.59% ± 11.23%) compared to the ZP group (22.25% ± 19.81%, *p* = 0.043). Given the non-significant omnibus interaction, this finding should be interpreted cautiously. For mean RT, the Group × Time interaction was not significant (Fgt(1.676,95.556) = 0.450, *p* = 0.604), and no significant main effect of Group was observed (Fg(1,57) = 2.021, *p* = 0.161). However, an exploratory analysis at 8 weeks showed significantly faster RT in the ZS group compared to the ZP group (F(1,57) = 4.143, *p* = 0.046).

**Table 3:** PVT parameters in patients with IDDS across the treatment period

Variable	Group	Baseline	4 Weeks	8 Weeks	Statistics	Post
Behavioral						
Mean RT, ms	ZS	0.44 ± 0.10	0.45 ± 0.10	0.42 ± 0.05	Fg(1,57) = 2.021, *p* = 0.161, η²=0.034 Ft(1.676,95.556) = 0.250, *p* = 0.740, η²=0.004 Fgt(1.676,95.556) = 0.450, *p* = 0.604, η²=0.008	ZS < ZP at 8 weeks F(1,57) = 4.143, p = **0.046**, η²=0.068
	ZP	0.46 ± 0.11	0.46 ± 0.09	0.47 ± 0.10		
Accuracy, %	ZS	95.62 ± 17.43	95.37 ± 17.89	98.65 ± 6.35	Fg(1,57) = 0.819, *p* = 0.369, η²=0.014 Ft(1.061,60.485) = 0.629, *p* = 0.440, η²=0.011 Fgt(1.061,60.485) = 1.127, *p* = 0.297, η²=0.019	n.s.
	ZP	98.65 ± 2.27	98.88 ± 1.98	98.32 ± 3.24		
Losses_Rate, %	ZS	4.38 ± 17.43	4.63 ± 17.89	1.35 ± 6.35	Fg(1,57) = 0.819, *p* = 0.369, η²=0.014 Ft(1.061,60.485) = 0.629, *p* = 0.440, η²=0.011 Fgt(1.061,60.485) = 1.127, *p* = 0.297, η²=0.019	n.s.
	ZP	1.35 ± 2.27	1.12 ± 1.98	1.68 ± 3.24		
Lapses Rate, %	ZS	16.20 ± 15.65	18.35 ± 17.20	13.59 ± 11.23	Fg(1,57) = 2.911, *p* = 0.093, η²=0.049 Ft(1.834,104.543) = 0.540, *p* = 0.569, η²=0.009 Fgt(1.834,104.543) = 0.515, *p* = 0.583, η²=0.009	ZS < ZP at 8 weeks F(1,57) = 4.301, p = **0.043**, η²=0.070
	ZP	22.65 ± 19.28	22.28 ± 21.04	22.25 ± 19.81		
N1, µV	ZS	-0.51 ± 0.68	-0.52 ± 0.59	-0.46 ± 0.40	Fg(1,57) = 0.310, *p* = 0.580, η²=0.005 Ft(1.829,104.242) = 2.244, *p* = 0.116, η²=0.038 Fgt(1.829,104.242) = 3.314, *p* = 0.044, η²=0.055	ZS < ZP at 8 weeks F(1,57) = 4.028, *p* = 0.050, η²=0.066
	ZP	-0.31 ± 0.56	-0.53 ± 0.84	-0.85 ± 0.99		
P3, µV	ZS	4.47 ± 2.04	4.21 ± 1.83	4.44 ± 1.64	Fg(1,57) = 0.228, *p* = 0.635, η²=0.004 Ft(2,114) = 0.406, *p* = 0.667, η²=0.007 Fgt(1.927,109.867) = 0.818, *p* = 0.440, η²=0.014	n.s.
	ZP	4.26 ± 2.20	4.24 ± 2.36	3.93 ± 2.33		

Notes: Data are mean amplitude (µV) ± SD. Fg, Ft, and Fgt represent F-values for the Group, Time, and Group × Time interaction effects, respectively; RT, reaction time; ERP, Event-Related Potential; ZS: Zolpidem + *Shugan Jieyu* (*n* = 30); ZP: Zolpidem + Placebo (*n* = 29); n.s. = not significant. Greenhouse-Geisser corrected degrees of freedom are reported where Mauchly’s test of sphericity was violated (*p* < 0.05)

**Fig. 2:**
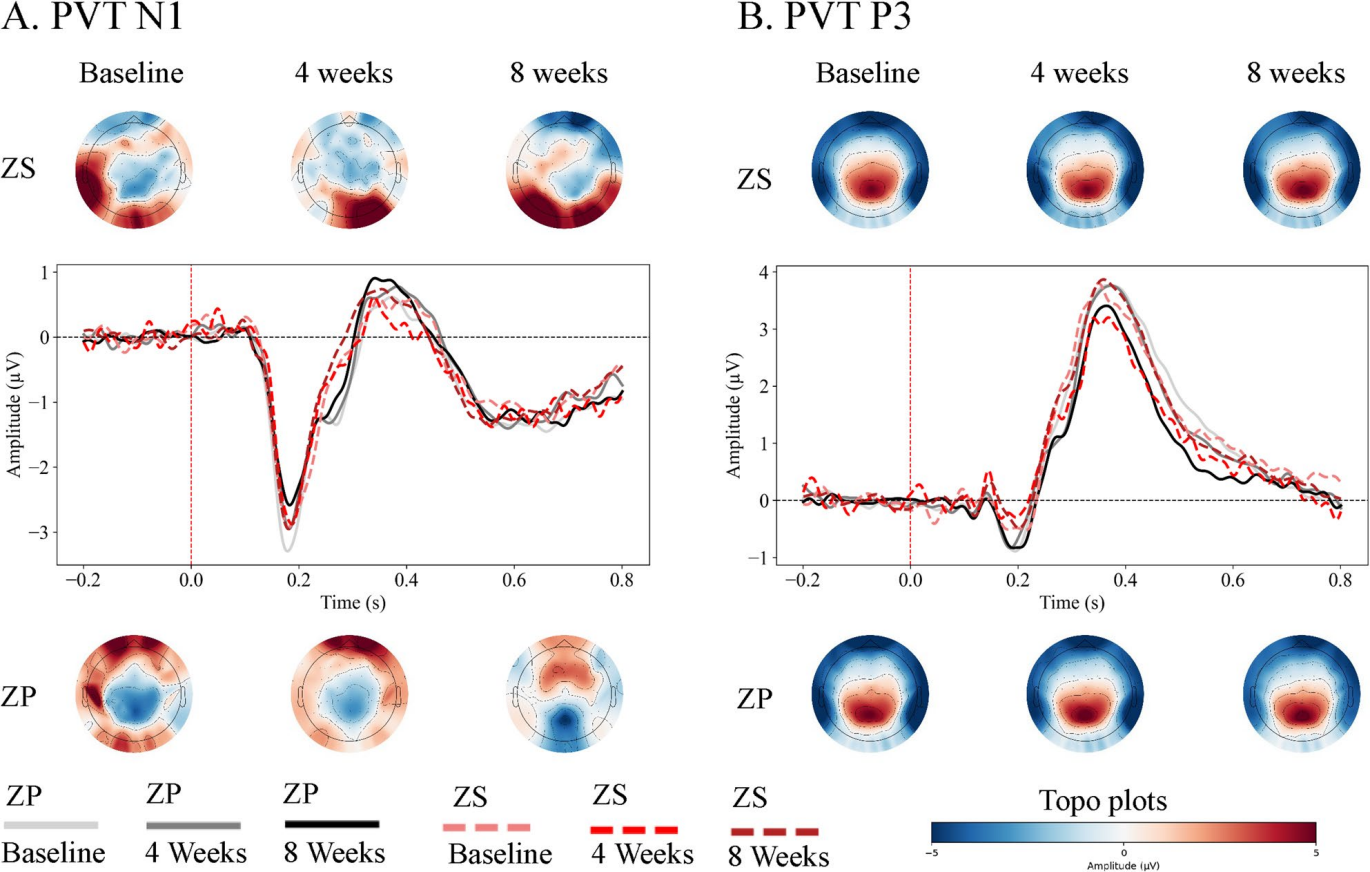
Event-Related Potential (ERP) waveforms and topographic maps for the Psychomotor Vigilance Task (PVT) across the treatment period. (**A**) Grand-average ERP waveforms for the N1 component, and corresponding topographic maps of the N1 mean amplitude distribution (50–150 ms) for the ZS (Zolpidem + *Shugan Jieyu*) and ZP (Zolpidem + Placebo) groups at Baseline, 4 Weeks, and 8 Weeks. (**B**) Grand-average ERP waveforms for the P3 component and topographic maps of the P3 mean amplitude distribution (300–600 ms). The topographic maps illustrate the scalp voltage distribution, with blue representing negative potentials and red representing positive potentials

Electrophysiological data from the PVT complemented these behavioral findings. A significant Group × Time interaction was found for the N1 amplitude (Fgt(1.829,104.242) = 3.314, *p* = 0.044). Post-hoc tests indicated that at the 8-week assessment, the ZS group demonstrated a trend of less negative N1 amplitude compared to the ZP group (F(1,57) = 4.028, *p* = 0.050). No significant effects were observed for the P3 component. The corresponding ERP waveforms and topographic maps for the N1 and P3 components are shown in Fig. 2 (Fig. 2).

## Discussion

This study found that the ZS therapy was associated with enhanced neural conflict monitoring at an early stage (reflected by a larger N2 amplitude at Week 4 on Go trials) and showed separate improvements in sustained vigilance at Week 8 (faster RT and fewer lapses on the PVT). These findings suggest that the combination therapy may confer benefits in two distinct cognitive domains, that is, cognitive control and sustained attention in patients with IDDS.

The dissociation between behavioral and electrophysiological outcomes in the Go/No-Go task is noteworthy. Although the overall Group × Time interaction for the N2 amplitude was not statistically significant, an exploratory analysis revealed that the ZS group showed a larger N2 amplitude on Go trials at Week 4 in the absence of concurrent behavioral differences. This finding should be interpreted cautiously. N2 amplitude in Go/No-Go tasks is considered a sensitive index of conflict monitoring and cognitive control, reflecting the engagement of frontal networks [10, 26, 27, 28]. The observation of enhanced N2 amplitude, even in the absence of behavioral changes, may tentatively suggest that the combination therapy could facilitate neural processes related to conflict detection [18, 40, 52]. However, given the exploratory nature of this finding, replication in larger samples is needed.

Regarding the P3 component, it is important to note that the parietal scalp distribution of our measurement (at Pz, P3, P4) aligns with the P3b subcomponent, which is traditionally linked to attentional resource allocation and context updating, rather than inhibitory execution per se [41]. No significant group effects were observed for the P3 component in either task. While a slight trend toward elevated P3 amplitude was visually observable in the ZS group, this did not reach statistical significance. This suggests that the primary impact of the combination therapy may be on earlier processes such as conflict monitoring (N2) and sensory gating (N1), rather than on later-stage attentional processes indexed by the P3b.

In addition, the ZS group showed better performance on the PVT task at Week 8, with faster RT and fewer lapses in attention. The PVT is a validated measure of sustained attention and is highly sensitive to neurobehavioral impairments due to sleep loss and hyperarousal [3, 31, 56]. The enhanced vigilance in the ZS group at Week 8 represents a separate and clinically meaningful benefit of the combination therapy. While both the N2 finding and the PVT improvement point toward enhanced cognitive function, they were observed in different domains (cognitive control vs. sustained attention) and at different time points. Future research should investigate whether these improvements are mediated by common mechanisms, such as reduced hyperarousal or improved sleep quality.

A significant Group × Time interaction was observed for the N1 amplitude. This finding appeared to be driven primarily by an increase in N1 negativity over time in the ZP group, while the ZS group’s N1 amplitude remained relatively stable. The N1 component is linked to early sensory processing and gating. If confirmed in future studies, a less negative N1 amplitude could indicate reduced hyperarousal and more efficient early sensory processing, potentially requiring fewer neural resources for basic stimulus perception [5, 13, 19, 53]. Such efficient resource allocation could theoretically support better behavioral performance, alleviating cognitive deficits associated with insomnia [5, 44]. Therefore, the combination helped mitigate the progression of sensory hyperarousal over the 8-week period, potentially preserving efficient early sensory processing. This stabilization of early sensory gating could theoretically support the improved sustained attention performance observed behaviorally in the ZS group at Week 8, although the between-group comparison at this single time point did not reach statistical significance (*p* = 0.050).

The combination of *Shugan Jieyu* and zolpidem may offer synergistic benefits. *Shugan Jieyu*’s purported effects on mood regulation via monoaminergic modulation could complement zolpidem’s sleep-promoting effects on the GABAergic system, potentially creating a neural environment more conducive to cognitive recovery. This synergy might involve the regulation of prefrontal networks involved in cognitive control and attention. By improving sleep and potentially reducing hyperarousal, the therapy could facilitate more efficient neural processing, as tentatively suggested by the ERP findings [15, 36]. However, the precise mechanisms remain speculative and require direct investigation in future studies using pharmacological neuroimaging approaches.

Clinically, these findings suggest that the *Shugan Jieyu* and zolpidem combination may offer cognitive benefits beyond sleep improvement in IDDS. The therapy was associated with enhanced conflict monitoring and improved sustained attention in two cognitive domains frequently impaired in patients with insomnia and depressive symptoms. These cognitive enhancements could potentially contribute to better functional outcomes and quality of life, though future studies should directly examine the relationship between these neurocognitive changes and real-world functioning.

This study has several limitations that should be acknowledged from our perspective. First, the relatively modest sample size, while comparable to many ERP studies, may limit the generalizability of the findings and preclude more sophisticated analyses like mediation. Future research should involve larger, more diverse cohorts to confirm and extend these results. Second, the follow-up period was relatively short; longer-term studies are needed to determine the durability of these cognitive benefits. Third, while we observed sequential neural and behavioral changes, our design does not permit causal inference. Future studies should employ mediation analyses or predictive modeling to formally test whether early ERP changes can predict later behavioral improvements. Finally, exploring other cognitive domains and employing additional neuroimaging methods would further elucidate the underlying neural mechanisms of this promising combination therapy.

## Conclusion

The findings from this randomized controlled trial demonstrate that the combination of *Shugan Jieyu* and zolpidem offers distinct benefits for cognitive function in patients with IDDS. The therapy was associated with an early enhancement of neural conflict monitoring, as evidenced by the N2 component, and later improvements in sustained vigilance, reflected in faster RT and fewer lapses on the PVT. These results suggest that this combination therapy is a promising intervention that may move beyond symptomatic sleep improvement to address specific neurocognitive deficits characteristic of IDDS.

## Data Availability

The data that support the findings of this study are available from corresponding author upon reasonable request.

## Abbreviations

AHI: Apnea-Hypopnea Index
ANOVA: Analysis of Variance
BMI: Body Mass Index
DSM-5: Diagnostic and Statistical Manual of Mental Disorders, Fifth Edition
EEG: Electroencephalography
ERP: Event-Related Potential
ICA: Independent Component Analysis
IDDS: Insomnia and Depressive Symptoms
MDD: Major Depressive Disorder
PHQ-9: Patient Health Questionnaire-9
PLMI: Periodic Limb Movement Index
PVT: Psychomotor Vigilance Task
RT: Reaction Time
SD: Standard Deviation
SPSS: Statistical Package for the Social Sciences
ZP: Zolpidem and placebo
ZS: Zolpidem and *Shugan Jieyu*
